# Retraction: A Rare Presentation of Isolated IgM Deficiency in a 28-Year-Old Male: A Case Report

**DOI:** 10.7759/cureus.r69

**Published:** 2023-01-26

**Authors:** Diana Voloshyna, Sidra Jamil, Syeda Iqra Mushir, Naila Akhter, Nimra Saleem

**Affiliations:** 1 School of Medicine, University of Michigan, Ann Arbor, USA; 2 Accident and Emergency, Glan Clwyd Hospital, Rhyl, GBR; 3 Pathology, Sir Salimullah Medical College, Dhaka, BGD; 4 Internal Medicine, Surgery, Radiology, Obstetrics and Gynecology, Federal Government Services Hospital Polyclinic, Islamabad, PAK; 5 Pathology, Shifa International Hospital, Islamabad, PAK; 6 Internal Medicine, Bangladesh Medical College and Hospital, Dhaka, BGD; 7 Internal Medicine, University of Health Sciences, Lahore, PAK

This article has been retracted due to academic fraud on the part of the authors, who submitted and published the work of their colleagues without their consent or knowledge. Both original authors of this article were excluded from the author list and have provided proof that they were the original authors. Last author Nimra Saleem obtained their draft and circulated it among a new group of co-authors before submitting and publishing it in Cureus. As such, the journal has made the decision to retract this article due to academic fraud led by last author Nimra Saleem.

